# Quantifying biological samples using Linear Poisson Independent Component Analysis for MALDI-ToF mass spectra

**DOI:** 10.1093/bioinformatics/btx630

**Published:** 2017-10-28

**Authors:** S Deepaisarn, P D Tar, N A Thacker, A Seepujak, A W McMahon

**Affiliations:** Division of Informatics, Imaging and Data Sciences, The University of Manchester, UK

## Abstract

**Motivation:**

Matrix-assisted laser desorption/ionisation time-of-flight mass spectrometry (MALDI) facilitates the analysis of large organic molecules. However, the complexity of biological samples and MALDI data acquisition leads to high levels of variation, making reliable quantification of samples difficult. We present a new analysis approach that we believe is well-suited to the properties of MALDI mass spectra, based upon an Independent Component Analysis derived for Poisson sampled data. Simple analyses have been limited to studying small numbers of mass peaks, via peak ratios, which is known to be inefficient. Conventional PCA and ICA methods have also been applied, which extract correlations between any number of peaks, but we argue makes inappropriate assumptions regarding data noise, i.e. uniform and Gaussian.

**Results:**

We provide evidence that the Gaussian assumption is incorrect, motivating the need for our Poisson approach. The method is demonstrated by making proportion measurements from lipid-rich binary mixtures of lamb brain and liver, and also goat and cow milk. These allow our measurements and error predictions to be compared to ground truth.

**Availability and implementation:**

Software is available via the open source image analysis system TINA Vision, www.tina-vision.net.

**Supplementary information:**

Supplementary data are available at *Bioinformatics* online.

## 1 Introduction

In mass spectrometry, the formation of gas phase ions from complex biomolecules typically destroys structures of interest. John B. Fenn and Koichi Tanaka overcame this problem, sharing the 2002 Nobel Prize in chemistry for matrix-assisted laser desorption/ionisation (MALDI) and electrospray ionisation (ESI), see Hillenkamp and Peter-Katalinić (2007). MALDI co-crystallizes complex samples within an easy to ionize matrix. Samples and matrix are vaporized and ionized with a laser, giving a pulsed source of ions ideal for ToF mass analysis. The ability to mass analyze large molecules with high detection sensitivity makes MALDI attractive for biological sample analysis, with applications ranging from milk adulteration detection, e.g. Calvano *et al.* (2013), to cancer studies, e.g. Rodrigo *et al.* (2014). MALDI can also form images by sampling across a 2D lattice, Fülöp *et al.* (2016), with mass peaks forming pixel values. These datasets are massively rich, with hundreds of mass-specific images able to be generated per acquisition. A method of data-mining such images would be a valuable enabling tool, allowing molecular correlations to be identified and mapped upon biological structures. Such a system must quantitatively model the complex variations and attribute them to classes of interest, e.g. tissue types. This may be achieved using linear modelling approaches, such as Independent Component Analysis (ICA), as in Gut *et al.* (2015). As a step towards a more general data-mining system, we present our own ICA approach that is believed to match well with the properties of MALDI data and so provide additional advantages over traditional linear modelling methods.

MALDI mass spectra (MS) are complex and highly variable. Careful preparation and acquisition can mitigate against some factors, e.g. Seeley *et al.* (2008), but requires training, practice and skill. Ideally, a homogeneous specimen might be expected to produce MS that are repeatable to levels of statistical sampling noise. However, MS exhibits many other modes of variation:

- Ionisation and detection vary depending upon local matrix density, chemistry, laser intensity and duration of acquisition, see Astigarraga *et al.* (2008).
- Long chain molecules can fragment by a number of mechanisms and also have isotopic variations.
- Protonation is the intended ionisation process, however, sodium and potassium ionisation is often observed, even following attempts to wash away soluble salts.
- Suppression effects exist, where the presence of certain chemicals can mask or change the appearance of others due to different affinities for attracting charge.
- Unwanted ions can contaminate mass spectra, including those from the MALDI matrix.
- The location of peaks (flight time) can differ between spectra if mass analysers are not calibrated for each acquisition. This is a function of surface geometry, as slight differences in a sample‘s height and local ion extraction fields correspond to slight differences in ToF.
- There is a near-continuous ‘chemical noise’, from un-gated post-source decay processes and ion scattering, superimposed on any inherent instrumentation noise.

A complex biomolecule will generate a series of MS features, which undergo correlated variations in intensity and position, depending upon equipment settings and local sample environment, e.g. Szájli *et al.* (2008). Aside from these variations, MALDI MS are approximately linear combinations of sub-spectra from a sample‘s constituent chemical components. Some sources of variation are reduced through pre-processing. Baseline corrections can remove background by subtracting a smooth function fitted beneath peaks, e.g. Williams *et al.* (2005). Alignment can be achieved by shifts of spectra, with various forms of interpolation applied for sub-bin precision, e.g. Jeffries (2005). Peak detection and integration can be achieved by thresholding, direct summation of *m*/*z* bins, or by the fitting of Gaussians, e.g. Yang *et al.* (2009). In previous work, we developed pre-processing methods for use where Poisson noise is dominant in peaks and Gaussian noise is dominant in background, see Seepujak *et al.* (2017).

A basic approach to MS analysis is to inspect single peaks that correlate maximally with sought measurements, e.g. Cahill *et al.* (2016). A peak can be normalized to a second peak (or integral over a region) in order to estimate relative compositions. Peaks may also be artificially added to act as internal standards, such as in Chumbley *et al.* (2016). Signal affected by high levels of ambiguity or confounding variability is thus excluded, at the cost of discarding potentially useful information. More efficient methods extract correlated peak variations, such as Principal Component Analysis (PCA) and Independent Component Analysis (ICA). These approximate data as weighted combinations of unit vectors, each representing correlated sets of peaks. Comparisons between linear models can be found in Gut *et al.* (2015) and Nicolaou *et al.* (2011), with evidence that ICA is most beneficial. The formulations of standard PCA and ICA algorithms are based upon uniform independent Gaussian errors, often conveniently leading to closed-form solutions. However, MALDI may not be compatible with these assumptions. In particular, Poisson statistics may better describe the counting of ions. Evidence of MALDI’s Poisson nature has been highlighted by others, e.g. Harn *et al.* (2015) and Piehowski *et al.* (2009), which we further investigate in this work.

**Fig. 1.**
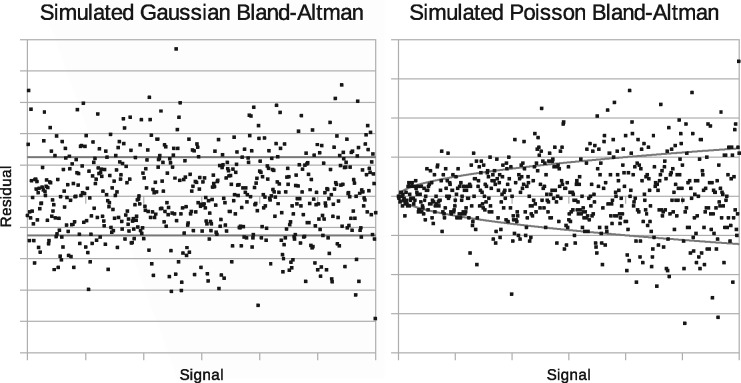
Monte Carlo generated Bland-Altman plots showing behaviour of uniform independent Gaussian noise (left) and independent Poisson noise (right)

The behaviour of noise can be assessed using Bland-Altman plots, see Bland and Altman (1986). These plot deviations from expected values as a function of signal strength. Figure 1 illustrates independent, identically distributed (iid) Gaussian noise, giving residuals with a fixed spread, and also Poisson noise, where residuals grow with the square-root of the signal. A square-root transform (Anscombe, 1948) can approximately convert Poisson noise into iid Gaussian noise, but this invalidates any assumed linear model of signal as a consequence. The main modelling options available and their key properties are listed in Table 1.

**Table 1.** Modelling options, with statistical and signal assumptions, available for varied data properties

Model	Noise	Signal	Orthogonal	Coefficients
PCA	iid Gaussian	∑αx	Yes	+/-
PCA with Anscombe	Poisson	$\sum \alpha \sqrt{x}$	Yes	+/-
ICA	iid Gaussian	∑αx	No	+/-
ICA with Anscombe	Poisson	$\sum \alpha \sqrt{x}$	No	+/-
Non neg ICA	iid Gaussian	∑αx	No	+ only
Poisson ICA*	Poisson	∑αx	No	+ only
MALDI data	≈*Poisson*	≈∑αx	*No*	*+ only*

*Note*: Our selected method (*) is based upon assumptions matching the properties of MALDI mass spectra. PCA (Jolliffe, 1986), ICA (Comon, 1994), Non neg ICA (Plumbley and Oja, 2004; Plumbley, 2003), Poisson ICA* (Tar and Thacker, 2014). Italics emphasize that MALDI data has the properties noted in the row above, i.e. the method we propose to use.

Using Bland-Altman analysis, we present evidence that a Gaussian noise assumption is inappropriate for MALDI data and that a Poisson assumption is more realistic. In recent work we derived an ICA method for data with Poisson sampling characteristics, see Tar and Thacker (2014): Linear Poisson Modelling (LPM). It has been applied to planetary and medical images (see Tar *et al.*, 2015, 2017). We believe this method [(*) in Table 1] provides the best match to the properties of MALDI data and is therefore evaluated here on the task of measuring mixtures of complex lipid specimens. Our method incorporates a Likelihood estimation procedure and a predictive error theory capable of assessing the effects of Poisson noise on measurements. An extension, ‘MAX SEP’, is designed to reduce degeneracy inherent in linear modelling, aiding interpretation of components allowing them to be attributed to biologically meaningful MALDI sub-spectra. The aim of MAX SEP is similar to that of the varimax, quartimax and equimax rotations, e.g. Kaiser (1958), but is appropriate for positive only data. This current study uses mixtures of cow‘s milk and goat‘s milk; lamb brain mixed with lamb liver extracts; and lamb white matter and grey matter, targeting mass ranges associated with the samples’ lipid content. In addition to applying our new LPM method, we perform single peak analyses on the same data to corroborate mixture measurements and compare attainable measurement precision.

## 2 Materials and methods

### 2.1 Sample preparation and MS acquisition

Binary mixtures in differing proportions (e.g. class A: class B) act as ground truth: cow milk with goat milk, chloroform extracts of homogenized lamb brain and liver and of lamb brain white matter and grey matter. For each pair, mixtures of lipids were carefully prepared in 11 proportions ranging from 0% A (100% B) to 100% A (0% B), in increments of 10%, with proportions determined by weight.

Milk mixtures were prepared prior to lipid extraction, whereas brain and liver lipids were extracted first, then mixed afterwards. For milk, 1 ml at each proportion underwent the lipid extraction procedure. For brain and liver tissues, lipid extraction was done separately using 2 g of homogenized tissue of each type. Lamb brain was cut in its coronal axis to approximately 1 cm^2^ for dissection of white and grey matter. Lipids were extracted by adding 2:1 methanol:chloroform (4.5 ml), chloroform (2 ml) and deionized water (1 ml) to the samples before mixing well. Samples were centrifuged at 1300 rpm for 2 min at 20 °C. Levels of natural salts in the resulting lipid extracts were reduced by the addition of 1 ml of deionized water before being centrifuged again.

10 mg/ml of 2,5-dihydroxybenzoic acid (LaserBio Labs) in acetonitrile with addition of 0.1% trifluoroacetic acid was prepared as a matrix solution. The matrix solution was mixed with a ratio of 1:1 for the milk and 3:2 for the tissue to form MALDI specimens. Double layers of a MALDI specimen, 1 microlitre each layer, were deposited onto a stainless steel MALDI target plate. 8 repeat depositions were applied per mixture proportion to provide repeatability data. The location of each proportion was randomly positioned on the target plate to avoid correlations between the spatial organisation and mixing amounts.

An AXIMA (curved-field reflectron time-of-flight) mass spectrometer, manufactured by Shimadzu Biotech, was used to acquire the MALDI MS data. Where the MALDI ionisation system of the instrument is a 349 nm neodymium-doped yttrium lithium fluoride (Nd: YLF) laser of <5 ns pulse width and approximately 200 Hz repetition rate. Using the positive reflectron mode, an ion extraction energy of up to 24 kV was allowed with an effective drift length of 2.0 m. The LAUNCHPAD proprietary software was used throughout all of the experiments for controlling acquisition. During acquisition, default settings were adopted (i.e. 200 profiles, 5 shots, pulsed extraction 750 Da, mass range up to 1500 Da). A total of 88 spectra were obtained for each mixture type, one for each of the deposited targets.

### 2.2 Pre-processing

Low *m*/*z* MS peaks (e.g. sodium and matrix ions) contain little information regarding lipid content. A mass window (*m*/*z*) between 650 and 850 was selected for milk mixtures; a window between 690 and 890 for brain: liver mixtures and white: grey matter. The total quantity of signal gathered per spectrum is difficult to control, making it necessary to pre-filter poor data. Spectra containing low signal within this window were rejected on grounds of low signal-to-noise. Spectra containing high signal were also rejected to avoid saturated peaks. A combination of visual inspection and goodness-of-fit tests determined which spectra were kept in order to build satisfactory models. This left 66 milk mixtures, 80 brain: liver mixtures and 82 white:grey matter mixtures, out of the original 88 per group.

Preprocessing is performed to ensure that data behaves as linearly additive histograms, with independent Poisson noise, as is required for LPMs to opperate correctly. The methods developed in Seepujak *et al.* (2017) achieve this. A peak alignment procedure is applied to minimize unwanted shifting of peaks. A baseline correction that assumes noise on the background is approximately Gaussian with zero mean is applied. Finally, histograms are produced containing only bins for significant peaks, with peaks integrated into each bin and inter-peak gaps removed. A total of 102 peaks were retained in the milk spectra, 76 peaks were retained in the brain: liver spectra and 67 in the white: grey matter mixtures.

### 2.3 Peak ratio analysis

We apply a simple peak-ratio analysis to estimate mixing proportions for the milk and brain: liver mixtures as a bench-mark against which the new LPM analysis can be compared. Within the lipid window, the largest peak is used for normalization. This peak is at *m*/*z* 760.5 in all three mixture cases, corresponding to a phosphocholine. At each mixing proportion, all peaks are divided by the reference peak, with results for each peak plotted against the known ground-truth proportions. A least-squares fit is computed for each peak, which should correlate (or anti-correlate) well with the ground truth if there is useful information present. The most informative peaks (providing smallest errors) were compared to the LP-ICA results. These peaks were at *m*/*z* 706.2 for milk mixtures, 786.5 for brain:liver and 734.5 for white:grey matter.

Sources of variability, including efficiency losses and possible contamination, makes it unlikely that a linear trend extracted from a single peak will have a slope and intercept that exactly predicts ground-truth. Rather, the fitted line is used to calibrate a linear predictor that maps normalized peaks to ground truth proportions. The standard deviation of predictions around the calibrated line is used as an estimate of the measurement accuracy attainable from each peak.

### 2.4 Linear Poisson ICA analysis

LPMs describe the shape and variability of distributions found within histograms using a linear combination of simpler fixed components, with Likelihood estimates of parameters (e.g. Barlow, 1989) using Expectation Maximisation. Each component can be viewed as a probability mass function (PMF) for a sub-spectrum, representing some correlated set of peaks. Unlike other linear models, LPMs use mixtures of PMFs, rather than unit vectors. This permits positive-only co-efficients, appropriate for counting applications such as ion counts in mass spectra. The mixture of components, fitted on a spectrum-by-spectrum basis, describes a spectrum as a weighted sum of sub-spectra as described in Eq. 1 of Supplementary Appendix S1. An LPM must determine the necessary PMFs (i.e. sub-spectra) required to describe the distribution of spectra. This process is a Poisson compatible form of ICA, or LP-ICA, which maximizes a Likelihood formulation of the problem (Eq. 2 of Supplementary Appendix S1). The number of components required to describe a set of spectra is determined through a model selection process that aims to reach a satisfactory $\chi_{D}^{2}$ goodness-of-fit (Eq. 3 of Supplementary Appendix S1). Satisfactory fits are those that either reach a minimum, or lie upon a plateau. In cases of a plateau, failing to achieving a true minimum is compensated for in the error theory, as error covariances are scaled by the final goddness-of-fit. Details of the full method and its validation in other applications can be found in Tar and Thacker (2014) and Tar *et al.* (2015, 2017).

#### *2.4.1* MAX SEP

The Likelihood estimates of LP-ICA components and weighting factors need not be unique. Due to the possibility of linear degeneracies there can be multiple equally good solutions. The MAX SEP algorithm can reduce this problem by manipulating components to increase their independence. Whilst this may not result in the best global solution, it allows the best to be selected from multiple local solutions. We argue that, given a choice between multiple equivalent Likelihood models, the better models are those which have better physical meaning.

What constitutes physical meaning is dependent upon the system being modelled. In the case of mass spectra, the components should map onto the correlated appearance of different chemicals associated with different types of biological sample. If this is achieved then model coefficients will be proportional to the quantities of different materials present, i.e. amount of brain or liver. However, the data fitting process guarantees only that the extracted linear model passes through a best-fit hyperplane; the LP-ICA components themselves are linearly degenerate. LP-ICA components may be linear combinations of the underlying biological samples. Typically, we might expect the components extracted to require modification (via subtraction of common structure) to remove unwanted components of the spectra.

In order to rectify this problem, it is reasonable to assume that certain chemicals will exist within some biological materials, but not others. This should result in some mass values being zero in one sample and finite in another. Subtracting the maximum amount of each LP-ICA component from all others increases the chance of finding unique stable solutions and makes model structure ‘simpler’. The criteria for defining components with simple structure were first suggested by Thurstone (1947) with respect to unit vector models in Factor Analysis, the first three of which:

- each row [data vector/histogram] contains at least one zero;
- for each column [factor/component], there are at least as many zeros as there are columns (i.e. number of factors kept);
- for any pair of factors, there are some variables with zero loadings on one factor and large loadings on the other factor;

are consistent without observation of mass spectra behaviour. The ‘loadings’ in unit-vector models are equivalent to histogram bins found within Linear Poisson Models. The ‘factors’ are components, equivalent to probability mass functions in LPMs. The MAX SEP algorithm attempts to achieve the ‘simple structure’ criteria by maximising the differences between PMF components to make them as separate and unique as possible. If a weighted amount of a PMF can be subtracted from another unweighted PMF, such that no probability goes below zero, then a new ‘PMF’, can be computed.

#### *2.4.2* Mapping components to classes

The mixtures of two different biological materials (e.g. brain, liver) will be considered to be composed from class A and class B. If spectra were stable to within the limits of Poisson sampling and if all molecules within samples were detected with 100% efficiency, we would expect to extract only 2 components from such mixtures, i.e. the spectrum for class A and the spectrum for class B. In practice, the numerous sources of variation noted in Section 1 lead to multiple components being required to describe spectra. Additionally, the ground-truth measurements are based upon the mass of total samples, which is not, strictly, what is being measured by the model coefficients. The fragmentation of molecules and their different affinities for attracting charge means only a small fraction of what is in a sample is ever detected, plus the windowing of data and thresholding of small peaks introduces further efficiency losses. As a consequence, the components and their quantities need further interpretation.

Firstly, components must be attributed to classes of material. A component may belong to class A, B, or be contamination belonging to neither class. Secondly, the relative efficiency with which components contribute to the total mass needs to be estimated. These can be solved by the introduction of a new weighting parameter for each component. These weights are optimized in order to achieve the best linear trend between sums of components and ground-truth. This is described in Eqs. 6 to 8 in Supplementary Appendix S1.

### 2.5 Spectra error analysis

The values recorded within spectra mass bins are expected to be Poisson in nature, as peak heights are proportional to ion counts which are discrete events occurring in time, consistent with a Poisson process. However, there are additional sources of noise, therefore the Poisson assumption must be checked. The residuals between spectra models and original spectra can be used to assess the validity of the assumption. If binned values are indeed Poisson in nature then the residuals should grow proportionally to the square-root of the bin quantity. Any fixed scaling of the Poisson process should too be revealed as a scaling factor on the square-root dependency. Bland-Altman plots, i.e. Bland and Altman (1986), can be constructed and a power-law error model fitted to assess both of these properties.

Bland-Altman plots are scatter plots which record expected bin values (i.e. expected peak intensity) on the *x*-axis versus deviations away from the expected values on the *y*-axis. The linear model predictions are used as estimates of expected bin values (*x*-axis), and residuals between model and spectra are the observed deviations (*y*-axis). A power-law function can be fitted to resulting plots to determine the behaviour.

### 2.6 Measurement error analysis

Sampling errors in spectra histograms combine to give a level of uncertainty on the estimated quantity measurements. In order to factor these uncertainties into final mixture proportions they must be propagated through the EM algorithm using error propagation, as described in Barlow (1989). This process uses derivative calculations to assess how small changes in inputs (i.e. Poisson noise in data) affects small changes in outputs (i.e. proportion measurements). Eqs. 10 to 11 of Supplementary Appendix S1 describe this process. Predicted errors via this method can be compared to true measurement errors by dividing predictions by the deviations seen against ground-truth. These form a Pull distribution, which if unbiased should have a mean of zero, and if precision is correctly predicted should have a width of unity.

**Fig. 2.**
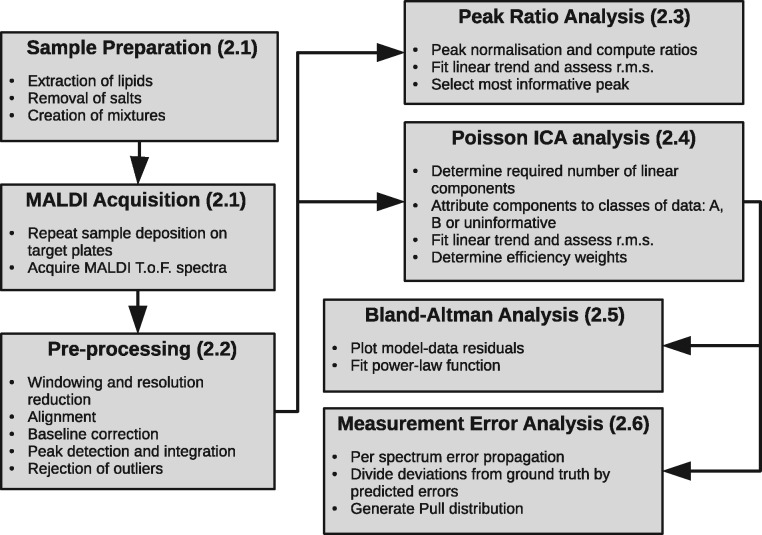
Processing work-flow diagram

In addition to the sampling errors, the Poisson ICA modelling processes is a numerical optimization method that utilizes random initializations leading to multiple local optima. Local solutions are similar, but do add a level of variability to results. To quantify this, multiple models (50) are built to assess the spread of solutions (Fig. 2).

## 3 Testings

**Fig. 3.**
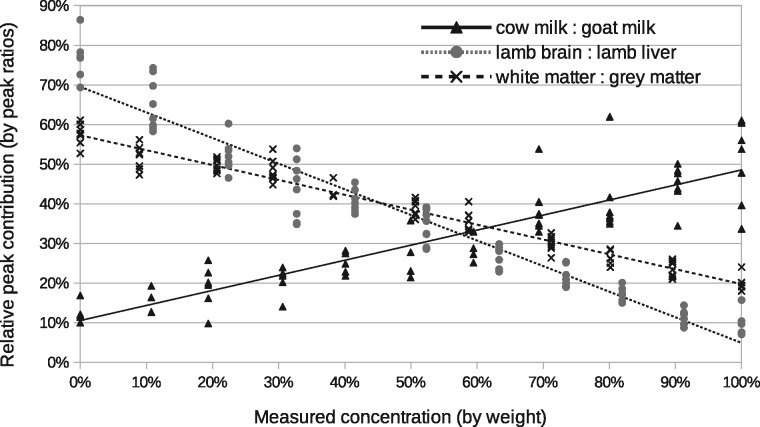
Correlation with ground-truth via peak ratio analysis: milk mixtures using *m*/*z* 706.2 peak normalized to *m*/*z* 760.5 peak; lamb tissue mixtures using *m*/*z* 786.5 peak normalized to *m*/*z* 760.5 peak; white: grey matter using *m*/*z* 734.5. The *x*-axis shows the ground-truth mixing proportions. Each cross is a peak ratio estimate from a different spectrum, with repeatability data at each 10% increment. Deviations from the fitted line (least square) show typical measurement accuracy

The peak ratio approach found that the peak at *m*/*z* 706.2 correlated best with changes in milk proportions, *m*/*z* 786.5 correlated best with changes in lamb tissue proportions, and 734.5 best for grey: white matter mixtures (Fig. 3). These peaks provide a relative measurement precisions of ±16%, ±8% and ±6%, respectively (Fig. 7).

**Fig. 4.**
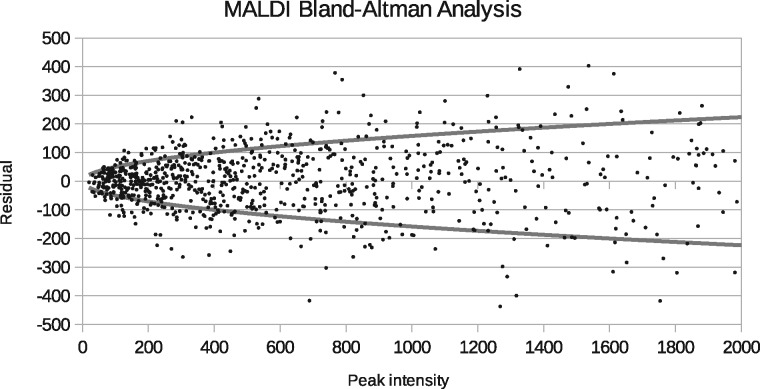
Bland-Altman plot showing behaviour of model residuals (*y*-axis) as function of peak intensity (*x*-axis). Each point represents a residual between an LP-ICA modelled spectrum bin and actual spectrum. The fitted curves (power law of Eq. 9) show ±1 standard deviation error as a function of peak intensity consistent with Poisson statistics

Bland-Altman analysis confirm that the pre-processed MALDI spectra are consistent with Poisson statistics (Fig. 4). The power-law growth parameter (*b* in Eq. 9 of Supplementary Appendix S1), was estimated as 1.04 ± 0.02, completely consistent with Poisson style growth in residuals as a function of peak intensity. This justifies the application of Linear Poisson Models to perform ICA and mixture quantitation. The power-law scaling parameter (*a* in Eq. 9) was estimated as 23.9 ± 1.4, consistent with the $\chi_{D}^{2}$ goodness-of-fit, suggesting that each Poisson event is equivelent to an increase of 5 units of signal intensity.

**Fig. 5.**
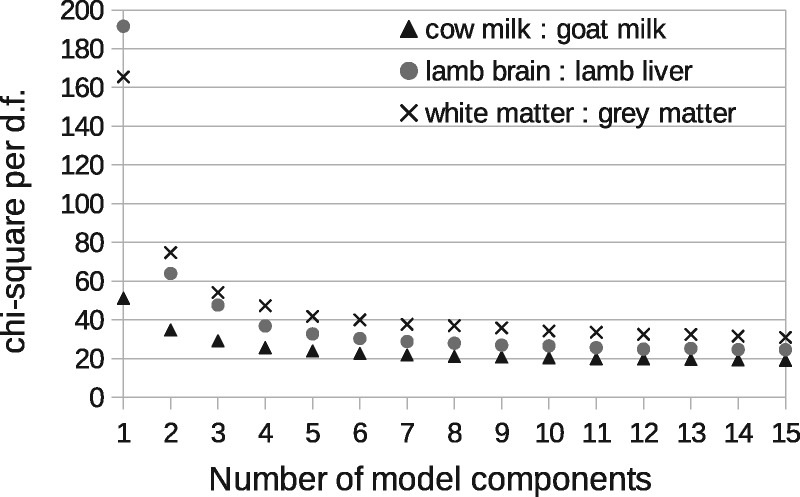
Determination of model order for LPMs. This curve shows the goodness-of-fit (Eq. 3 of Supplementary Appendix S1) of LP-ICA models as a function of the number of model components, where each component represents a sub-spectrum that is a mode of correlated spectra variation

**Fig. 6.**
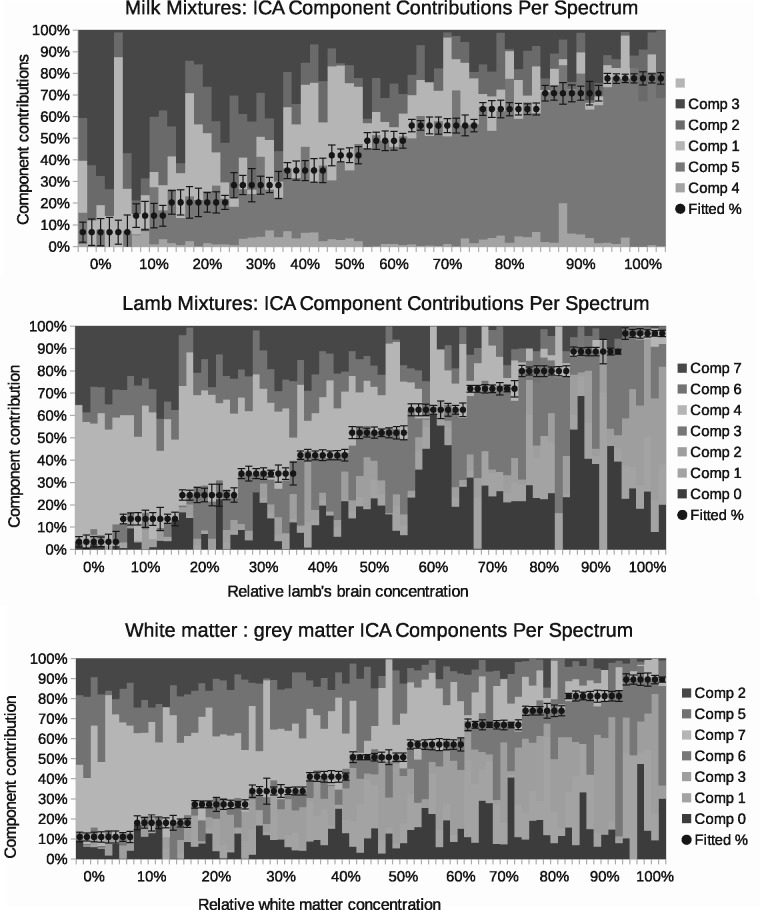
Composition of spectra in terms of weighted contributions of extracted LP-ICA components. Each 10% increment is shown as a step, where each step contains repeatability data for independent spectra with the same mixing proportions. The dots show the best fitted trend. Each bar shows the relative proportion of each LP-ICA component present within a spectrum. The error bars are the Linear Poisson Model predicted errors. The components ‘comp 1’ etc. are listed in the keys from top to bottom in the same order as they appear in the figure

**Fig. 7.**
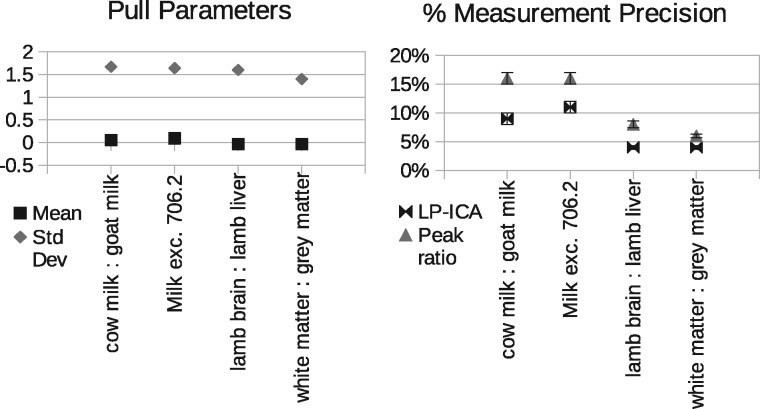
Left: Predictive ability of LPM error theory, as measured using pull distributions. A pull distribution should have a mean of zero and standard deviation of unity if predicted errors match observed errors. This is achieved in all variants of our experiments. Right: Measurement precision of peak ratio analysis versus LP-ICA analysis. Values are 1 standard deviation relative errors, expressed as percentage of quantity measurements. LP-ICA method is more precise in all experiments

A total of six components were found to be required to sufficiently model the milk spectra, at which point the goodness-of-fit begins to plateau (Fig. 5). The lamb brain:liver spectra required eight and white:grey matter also required eight. Once attributed to sample classes, one milk component, one brain:liver component and one white:grey component were rejected as being uninformative (due to contamination or ambiguity), with the remaining compositions showing a clear linear trend against known mixtures (Fig. 6). These provided relative measurement precision of around ±9, ±4 and ±4%, approximately doubling that attained via peak ratio analysis (Fig. 7). Even when the peak known to correlate best with milk mixtures was removed from the LP-ICA analysis, a precision of ±11% could be achieved. The spectra components for milk can be seen in Supplementary Appendix S2.

Pull distribution analysis (actual deviations from ground-truth divided by predicted deviations) show that LP-ICA model measurements are unbiased (mean consistent with zero) and predicted errors successfully describe the majority of measurement noise, with true errors being 1.6 times larger than predicted (Fig. 7).

## 4 Discussion

Two alternative methods to making quantitative measurements from MALDI mass spectra of biological samples have been presented: peak ratios and Linear Poisson ICA analysis. Both mitigate against confounding variability (caused by local matrix density, chemistry, ionisation field, etc.) and also ambiguity (caused by common molecular constituents in different samples) using very different approaches. The former avoids problems by simply discarding mass peaks which are adversely affected, selecting those which empirically correlate best with sought measurements. The latter is far more sophisticated, modelling sources of variability, learning correlations between any number of peaks and attributing them to meaningful classes of data. This latter method is far more efficient, as much more signal is retained. The peak ratio method uses only 14% of the total signal available in the preprocessed lamb spectra, whereas the LP-ICA approach uses 90%, which immediately should provide an advantage through sample size alone. The LP-ICA approach achieves levels of measurement precision double that attainable through peak ratio analysis, with 1 standard deviation errors reducing from ±16 and ±8% to as small as ±9 and ±4%, for milk and lamb tissue mixtures, respectively. Achieving this increased precision using the peak ratios method would require at least quadruple the quantity of data (assuming errors fall with the square-root of sample size). Grey: white matter measurement errors reduced from ±6 to ±4%, suggesting that most information is already extracted from the single peak at *m*/*z* 734.5.

The efficient use of data is perhaps best illustrated by the LP-ICA method when the most informative milk peak (*m*/*z* 706.2) is removed. Despite the ambiguity of remaining peaks, measurements could still be made using the ICA method with errors of ±11%. If the inverse variance is used as a measure of information content, the LP-ICA analysis precision using all milk peaks (±9%) is consistent with combining the all-but-one analysis (±11%) with the conventional peak ratio results from the 706.2 peak (±16%).

In addition to the increased precision gained using the new method, the LPM error theory (Section 2.6) provides the capability to predict measurement errors on a spectrum-by-spectrum basis. These predictions explain the majority of measurement noise, as confirmed by Pull distributions which should have a mean of zero and width of unity. Figure 7 shows that there is no bias (mean consistent with zero) and that true errors (assessed against ground-truth) are close to those predicted. Error predictions within a factor of 2 are generally deemed sufficient for scientific use, e.g. Barlow (1989) and Flannery (2009). As these errors are predictable from the input data, they do not require ground-truth to be computed. These predictive powers provide several advantages, permitting goodness-of-fits to be constructed, such as $\chi^{2}$, and revealing data-specific errors (see variable-sized error bars in Fig. 6). In contrast, the peak ratio method uses empiricism alone to determine measurement precision. This provides a single error estimate, the use of which relies upon an assumption of uniform errors across all spectra, which logically should not be the case due to differences in normalization. Furthermore, alternative analyses, such as PCA or conventional ICA do not provide error *predictions*, and must also rely upon empiricism and ground-truth.

Despite the success of the error predictions, observed errors were still larger than expected. The additional sources of unpredictable error include: the spread of local minima in the numerical ICA solutions; the Gaussian measurement noise superposed upon the Poisson sampling process; non-linearity; the potential need for a greater number of linear components; and imperfect preprocessing. The effect of local optima was assessed by making 50 attempts to build ICA models. The typical (median) precision attainable for brain:liver mixtures was ±4.59%, for milk mixtures the typical precision was ±9.77% and for white:grey matter ±5.15%. The best local solutions found were ±3.96% and ±9.05%, from brain:liver and milk respectively, which are the solutions used in the associated figures. Rather than relying upon a single model (i.e. one local ICA solution) a mean linear mixing prediction can be made from many local solutions, thereby reducing variability. The mean predictions from the 50 model attempts provide precisions of ±3.77% (brain:liver), ±8.72% (milk) and ±4.19% (white:grey).

The Poisson sampling assumption was validated via Bland-Altman analysis, showing that errors grow with the square-root of peak intensity, with an overall scaling factor of 24 (Fig. 4). The scaling factor measured is also consistent with the $\chi^{2}$ per degree of freedom of ICA models (i.e. 24 plateau reached in Fig. 5). Alternative modelling approaches, such as PCA, and other ICA methods, would be inappropriate due to their Gaussian noise assumptions and lack or predictive error theories.

If the MS acquisition pipeline was ideal, there would only be two sources of variability: changes in signal due to changing mixture proportions; and random Poisson sampling noise. Despite best efforts to homogenize mixtures, wash away salts and perform basic preprocessing, resulting spectra still contain numerous modes of variation. Six linear components were required to model milk and eight to model lamb tissue. Within these components, one was rejected from each model on account of its inability to provide information regarding mixture proportions. This could be due to either the component representing contamination, or the component could contain common structure indistinguishable between the sample classes. The remaining components are presumed to be modelling those sources of variation noted in Section 1, i.e. fragmentation, ionisation modes, isotopic variations etc. The number of components required to model the data could potentially be used as a measure of data quality. Preparation, acquisition and preprocessing steps could be optimized to minimize the number of required components.

Despite the numerous components required to describe the data, the MAX SEP algorithm provides the ability to attribute Poisson sampled components physical meaning, allowing their quantities to be used for measurement. The attribution of component quantities to classes of tissue are only valid if this physical meaning can be established. An alternative approach, based upon PCA or factor rotations for example, would not have been appropriate due to enforced orthogonality and non-physical negative weightings.

Finally, as a tool for future MALDI image data-mining, we believe the LPM approach could improve the information content of images by replacing pixels based upon peaks to pixels based upon LP-ICA component weights. Such images are expected to have better signal-to-noise, as pixels would incorporate information from many peaks. They may also better correlate with tissue types, giving a higher-level interpretation than simply being a map of specific chemicals. And finally, through the LPM error theory, pixels values are made quantitatively meaningful. The ability to quantitatively assess correlated chemicals and map them onto biological structures will be the focus of future work.

## 5 Conclusion

We have shown that a Linear Poisson Model analysis of MALDI mass spectra provides improved quantitative accuracy for the measurement of biological samples when compared to a conventional single-peak approach. There are only a relatively small number of peaks which are applicable to a single-peak analysis, as most are adversly affected by high levels of uncontrolled variability. LPMs successfully model this variability, permitting information in any number of peaks to be included in measurement estimation. The accuracy of milk, brain and liver mixture proportion measurements were doubled using our new approach.

In addition, the modes of variation found within MALDI mass spectra, in terms of sub-spectra combinations, can now be extracted and analyzed providing physically interperatable lower-parameter models, marking a step improvement in related ICA work in the field. The high levels of variability, sources of ambiguity and lack of error predictions also suggests that simple single peak ratios are unlikely to be quantitatively trustworthy. The approach we have demonstrated will be extended in future work to data-mine MALDI images.

## Supplementary Material

Supplementary Data

## References

[btx630-B1] Anscombe F. (1948) The transformation of Poisson, binomial and negative-binomial data. Biometrika, 35, 246–254.

[btx630-B2] Astigarraga E. et al (2008) Profiling and imaging of lipids on brain and liver tissue by matrix-assisted laser desorption/ionization mass spectrometry using 2-mercaptobenzothiazole as a matrix. Anal. Chem., 80, 9105–9114. 18959430 10.1021/ac801662n

[btx630-B3] Barlow R. (1989) Statistics: A Guide to the Use of Statistical Methods in the Physical Sciences. John Wiley and Sons, West Sussex, England, p. 55.

[btx630-B4] Bland J., Altman D. (1986) Statistical methods for assessing agreement between two methods of clinical measurement. Lancet, 327, 307310. 2868172

[btx630-B5] Cahill J. et al (2016) Absolute quantitation of propranolol from spatially distinct 20- and 40-µm dissections of brain, liver, and kidney thin tissue sections by laser microdissection-liquid vortex capture-mass spectrometry. Anal. Chem., 88, 6026–6034. 27214103 10.1021/acs.analchem.6b01155

[btx630-B6] Calvano C. et al (2013) MALDI-ToF mass spectrometric determination of intact phospholipids as markers of illegal bovine milk adulteration of high-quality milk. Anal. Bioanal. Chem., 405, 1641–1649. 23232957 10.1007/s00216-012-6597-z

[btx630-B7] Chumbley C. et al (2016) Absolute quantitative MALDI imaging mass spectrometry: a case of rifampicin in liver tissues. Anal. Chem., 88, 8920–23922398. 26814665 10.1021/acs.analchem.5b04409 PMC5080977

[btx630-B8] Comon P. (1994) Independent component analysis – a new concept? Sig. Process., 36, 287–314.

[btx630-B9] Flannery B.P. et al (2009) Numerical Recipes in c, Vol. 2 Cambridge University Press, New York, NY, USA, p. 660.

[btx630-B10] Fülöp A. et al (2016) Molecular imaging of brain localization of liposomes in mice using MALDI mass spectrometry. Nature, 6, 33791. 10.1038/srep33791 PMC5030664 27650487

[btx630-B11] Gut Y. et al (2015) Application of chemometric algorithms to MALDI mass spectrometry imaging of pharmaceutical tablets. J. Pharm. Biomed. Anal., 105, 91–100. 25543287 10.1016/j.jpba.2014.11.047

[btx630-B12] Harn Y. et al (2015) Deconvolving molecular signatures of interactions between microbial colonies. Bioinformatics, 31, 142–150. 10.1093/bioinformatics/btv251 PMC4765860 26072476

[btx630-B13] Hillenkamp F., Peter-Katalinić J. (2007) MALDI Mass Spectrometry Instrumentation, In MALDI MS: A Practical Guide to Instrumentation, Methods and Applications F. Hillenkamp and J. Peter-Katalinić (ed), Wiley-VCH Verlag GmbH & Co. KGaA, Weinheim, Germany. doi:10.1002/9783527610464.ch2.

[btx630-B14] Jeffries N. (2005) Algorithms for alignment of mass spectrometry proteomic data. Bioinformatics, 21, 3066–3073. 15879456 10.1093/bioinformatics/bti482

[btx630-B15] Jolliffe I. (1986) Principle Component Analysis. Springer, New York and Berlin.

[btx630-B16] Kaiser H.F. (1958) The varimax criterion for analytic rotation in factor analysis. Psychometrika, 23, 187–200.

[btx630-B17] Nicolaou N. et al (2011) MALDI-MS and multivariate analysis for the detection and quantification of different milk species. Anal. Bioanal. Chem., 399, 3491–3502. 21298416 10.1007/s00216-011-4728-6

[btx630-B18] Piehowski P. et al (2009) Time-of-flight secondary ion mass spectrometry imaging of subcellular lipid heterogeneity: Poisson counting and spatial resolution. Anal. Chem., 81, 5593–5602. 19530687 10.1021/ac901065s PMC2758657

[btx630-B19] Plumbley M. (2003) Algorithms for nonnegative independent component analysis. IEEE Trans. Neural Netw., 14, 534–543. 18238037 10.1109/TNN.2003.810616

[btx630-B20] Plumbley M., Oja E. (2004) A nonnegative pca algorithm for independent component analysis. IEEE Trans. Neural Netw., 15, 66–76. 15387248 10.1109/TNN.2003.820672

[btx630-B21] Rodrigo M. et al (2014) MALDI-ToF MS as evolving cancer diagnostic tool: a review. J. Pharm. Biomed. Anal., 95, 245–255. 24699369 10.1016/j.jpba.2014.03.007

[btx630-B22] Seeley E. et al (2008) Enhancement of protein sensitivity for MALDI imaging mass spectrometry after chemical treatment of tissue sections. J. Am. Soc. Mass Spectrom., 19, 1069–1077. 18472274 10.1016/j.jasms.2008.03.016 PMC2582528

[btx630-B23] Seepujak A. et al (2017) The statistical properties of raw and preprocessed ToF Mass Spectra, Internal Report, TINA Memos, 2016-007, University of Manchester.

[btx630-B24] Szájli E. et al (2008) Investigating the quantitative nature of MALDI-ToF MS. Mol. Cell. Proteomics, 7, 2410–2418. 18653768 10.1074/mcp.M800108-MCP200

[btx630-B25] Tar P., Thacker N. (2014) Linear Poisson models: a pattern recognition solution to the histogram composition problem. Ann. BMVA, 2014, 1–22.

[btx630-B26] Tar P. et al (2015) Automated quantitative measurements and associated error covariances for planetary image analysis. Adv. Space Res., 56, 92–105.

[btx630-B27] Tar P. et al (2017) Estimating false positive contamination in crater annotations from citizen science data. Earth Moon Planets, 119, 47–63. 10.1007/s11038-016-9499-9 PMC7114961 32269395

[btx630-B29] Thurstone L.L. (1947) Multiple-factor Analysis, University of Chicago Press, Chicago, US, ISBN-10 0226801098, ISBN-13 978-0226801094.

[btx630-B30] Williams B. et al (2005) An algorithm for baseline correction of MALDI mass spectra. In: *Proceedings of the 43nd Annual Southeast Regional Conference, 2005, Kennesaw, Georgia, Alabama, USA*, *March 18–20, 2005*, 1.

[btx630-B31] Yang C. et al (2009) Comparison of public peak detection algorithms for maldi mass spectrometry data analysis. BMC Bioinformatics, 10, 4. 19126200 10.1186/1471-2105-10-4 PMC2631518

